# Relation Extraction in Biomedical Texts Based on Multi-Head Attention Model With Syntactic Dependency Feature: Modeling Study

**DOI:** 10.2196/41136

**Published:** 2022-10-20

**Authors:** Yongbin Li, Linhu Hui, Liping Zou, Huyang Li, Luo Xu, Xiaohua Wang, Stephanie Chua

**Affiliations:** 1 School of Medical Information Engineering Zunyi Medical University Zunyi China; 2 Faculty of Computer Science and Information Technology University Malaysia Sarawak Sarawak Malaysia

**Keywords:** biomedical relation extraction, deep learning, feature combination, multi-head attention, additive attention, syntactic dependency feature, syntactic dependency graph, shortest dependency path

## Abstract

**Background:**

With the rapid expansion of biomedical literature, biomedical information extraction has attracted increasing attention from researchers. In particular, relation extraction between 2 entities is a long-term research topic.

**Objective:**

This study aimed to perform 2 multiclass relation extraction tasks of Biomedical Natural Language Processing Workshop 2019 Open Shared Tasks: relation extraction of Bacteria-Biotope (BB-rel) task and binary relation extraction of plant seed development (SeeDev-binary) task. In essence, these 2 tasks are aimed at extracting the relation between annotated entity pairs from biomedical texts, which is a challenging problem.

**Methods:**

Traditional research methods adopted feature- or kernel-based methods and achieved good performance. For these tasks, we propose a deep learning model based on a combination of several distributed features, such as domain-specific word embedding, part-of-speech embedding, entity-type embedding, distance embedding, and position embedding. The multi-head attention mechanism is used to extract the global semantic features of an entire sentence. Meanwhile, we introduced a dependency-type feature and the shortest dependency path connecting 2 candidate entities in the syntactic dependency graph to enrich the feature representation.

**Results:**

Experiments show that our proposed model has excellent performance in biomedical relation extraction, achieving *F*_1_ scores of 65.56% and 38.04% on the test sets of the BB-rel and SeeDev-binary tasks. Especially in the SeeDev-binary task, the *F*_1_ score of our model is superior to that of other existing models and achieves state-of-the-art performance.

**Conclusions:**

We demonstrated that the multi-head attention mechanism can learn relevant syntactic and semantic features in different representation subspaces and different positions to extract comprehensive feature representation. Moreover, syntactic dependency features can improve the performance of the model by learning dependency relation between the entities in biomedical texts.

## Introduction

### Background

Information extraction (IE) [1] involves extracting specific events or related information from texts; automatically classifying, extracting, and reconstructing useful information from massive amounts of content; and transforming it into structured knowledge. With the increasing demand for text mining technology to locate key information in biomedical literature, biomedical IE [2,3] has become a new research hot spot. Simultaneously, with the explosive development of biomedical literature, many research directions for biomedical IE have been promoted, such as named entity recognition, protein relation extraction [4], and drug interaction extraction [5]. In particular, it is a challenging and practical problem to detect the relation between annotated entities in the biomedical text under relation constraints, which is an important research direction.

The Biomedical Natural Language Processing Workshop-Open Shared Task (BioNLP-OST) series [6] is representative of biomolecular IE, which aims to facilitate the development and sharing of biomedical text mining and fine-grained IE. BioNLP-OST has made a great contribution to the development of biomedical IE and has been held for 5 times. The research topics of BioNLP-OST include fine-grained event extraction, biomedical knowledge base construction, and other scopes. This study mainly focused on the relation extraction of Bacteria-Biotope (BB-rel) task and the binary relation extraction of plant seed development (SeeDev-binary) task in BioNLP-OST 2019 [7]. These 2 multiclass subtasks are essential for predicting whether and what relationship exists between 2 annotated entities. This study contributes to the development of practical applications for biomedical text mining.

A series of innovative systems have achieved good results and actively promoted the development of biomedical IE. For example, in BB-rel and SeeDev-binary tasks, traditional relation extraction models are mainly based on feature-based [8,9] and kernel-based methods [10,11]. These methods rely on domain-specific knowledge or language tools to extract artificial features. For example, in the study by Björne and Salakoski [12], a relation extraction system was constructed using a feature based on the shortest dependent path and support vector machine (SVM). In recent years, deep learning (DL) models have been successfully applied in many fields of natural language processing, requiring less feature engineering and automatic learning of useful information from corpus data (Kumar, S, unpublished data, May 2017). In the biomedical relation extraction field, several well-known DL models have been gradually applied and have achieved excellent performance, including distributed representation [13,14], convolutional neural network (CNN) [15-17], and recurrent neural network [18-20]. Consequently, instead of complicating handcrafted feature engineering, we used the DL method to extract relations in biomedical texts.

The combined application of the distributed features of a full sentence is the most common method for biomedical relation extraction [13,21,22]. Here, we use a variety of distributed features, such as domain-specific word embedding [23], part of speech (POS) embedding [24], entity-type embedding [13], and distance embedding [25]. However, the commonly used model is difficult to focus on the key information of full sentence; therefore, the attention mechanism [26] has been proposed and proven to be successful in a wide range of natural languages processing fields, such as machine translation, reading comprehension, and sentiment classification [27-29]. In our proposed model, we use the multi-head attention mechanism proposed by Vaswani et al [30] to deal with the combination of distributed features of the full sentence. Multi-head attention can ignore the distance between words, directly calculate the dependency between words, and learn the syntactic and semantic features of sentences in different representation subspaces. We also constructed position embedding (PE) to inject position information to take advantage of the order of words in a sentence.

In our proposed model, we also integrated the shortest dependency path and dependency-type feature based on the syntactic dependency graph as one of the input features, which has been proven to be effective in several studies [19,31,32]. Although syntactic dependency features contain valuable syntactic information to facilitate the extraction of biomedical relations, they may still lose important information, such as prepositions before or after entities are likely to be discarded on the dependency path, which should play a key role [33]. Hence, this study adopts the combination of distributed features and syntactic dependency features as the final feature representation of biomedical texts, in which syntactic dependency features exist as supplementary features.

In this paper, we introduce a DL model to solve 2 biomedical relation extraction tasks: SeeDev-binary and BB-rel. We combined several distributed features and a multi-head attention mechanism to automatically extract global semantic features from long and complicated sentences. Syntactic-dependent features were also integrated into the model. As the shortest dependency path connecting 2 entities is short and concise, we apply a CNN to learn its features. We conducted extensive experiments, and our approach achieved *F*_1_ scores of 65.56% and 38.04% on BB-rel and SeeDev-binary tasks and achieved state-of-the-art performance on the SeeDev-binary task.

### Related Work

The BB-rel task was conducted 3 times [34] before, and the fourth edition [35] in the BioNLP-OST 2019 focused on extracting information about bacterial biotopes and phenotypes, motivated by the importance of knowledge on biodiversity for theoretical research and applications in microbiology, involving entity recognition, entity normalization, and relation extraction. This edition has been extended to include a new entity type of *phenotype*, relation category of *Exhibits*, and new documents. We mainly studied one of the subtasks, the relation extraction task (BB-rel), which is to predict the relationship of *Lives_In* category between *microorganism*s, *habitat*s, and *geographic* entities, and the relation of *Exhibits* category between *microorganism* and *phenotype* entities from PubMed abstracts and full-text excerpts, where entity annotation has been provided. Many researchers have contributed their efforts to the BB-rel task and have proposed innovative methods. For example, in Biomedical Natural Language Processing Workshop 2016, TurkuNLP team used the method of the shortest dependent path using the Turku event extraction system (TEES) [12] and 3 long short-term memory (LSTM) units, achieving an *F*_1_ score of 52.10% [31]. The bidirectional gated recurrent unit-Attn team proposed a bidirectional gated recurrent unit with an attention model, with an *F*_1_ score of 57.42% [36]. Amarin et al [33] combined feature combinations with an attention model and contextual representations to achieve a state-of-the-art performance with an *F*_1_ score of 60.77%. In BioNLP-OST 2019, almost all researchers used neural network models in various architectures. For instance, the Yuhang_Wu team used a multilayer perceptron and achieved an *F*_1_ score of 60.49% on the test set. The highest *F*_1_ score was 66.39%, which was submitted by the whunlp team [37]. They constructed a dependency graph based on lexical association, and used bidirectional LSTM (BiLSTM) [38] and an attention graph convolution neural network to detect the relation. In addition, the AliAI team innovatively used a multitask architecture similar to *Bidirectional Encoder Representations from Transformers* (BERT) and achieved 64.96%, which effectively alleviated the lack of information in the domain-specific field [39].

The SeeDev task [40] aims to facilitate the extraction of complex events on regulations in plant development from scientific articles, with a focus on events describing the genetic and molecular mechanisms involved in *Arabidopsis thaliana* seed development. The SeeDev task involves extracting 21 relation categories, involving 16 entity types, to accurately reflect the complexity of the regulatory mechanisms of seed development, which is a major scientific challenge. SeeDev was originally proposed at BioNLP-OST 2016 [6], and in 2019, the evaluation methodology focused more on the contribution of biology. It includes full and binary relation extraction, in which we mainly study the binary relation extraction subtask SeeDev-binary. To address this problem, most researchers have used traditional supervised machine learning approaches. These systems design artificial templates or manually extract many features based on domain-specific knowledge, such as linguistic features, semantic features, and syntactic information, which are added to the system as feature representations. Kernel-based machine learning algorithms such as SVM and Bayesian are then used to detect the relation categories, which are widely used for IE. For instance, the UniMelb team [41] developed an event extraction system using rich feature sets and SVM classifiers with a linear kernel. In addition, the MIC-CIS team [42] used an SVM combined with linguistic features to achieve optimal results on BioNLP-OST 2019. As the DL model gradually became the main research method, the DUTIR team [13] innovatively used a DL model based on distributed features and a CNN model [15]. The YNU-junyi team [14] integrated the LSTM model [18] based on a CNN model to address the problem that CNN alone cannot capture the long-range dependence of sequences, and they obtained an *F*_1_ score of 34.18% on the SeeDev-binary task of BioNLP-OST 2019.

## Methods

### Overview

**Figure 1:**
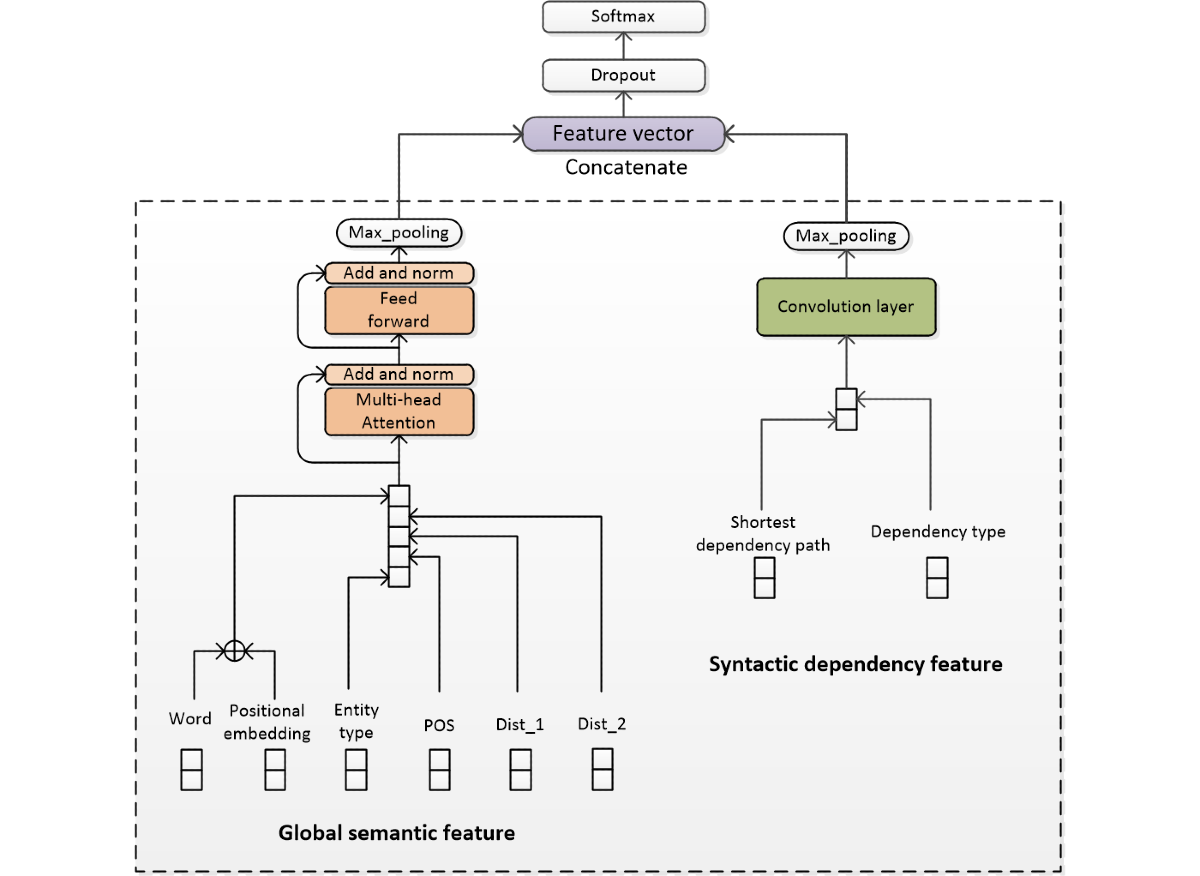
The overall architecture of our proposed model with global semantic feature based on feature combination and multi-head attention as well as syntactic dependency feature. Dist_1: distance embedding corresponding to the first entity in a sentence; Dist_2: distance embedding corresponding to the second entity in a sentence; entity type: entity type embedding; POS: part-of-speech embedding; Word: word embedding.

In this section, we describe our proposed model for the 2 biomedical relation extraction tasks in detail. The overall architecture is shown in Figure 1. The preprocessing of the data sets is described in the first part. In the second part, we introduce a series of distributed semantic features used in our method, and the multi-head attention mechanism used on them is introduced in the third part. The fourth part explains the construction of the syntactic dependency feature. In the fifth part, we introduce the classification and training details. Finally, we present the training and hyperparameter settings.

### Data Preprocessing

In the data preprocessing phase, we used TEES [12,31] to run a text preprocessing pipeline. The TEES system splits the text into sentences using the GENIA Sentence Splitter [43] and parses the sentences through the integrated the Brown Laboratory for Linguistic Information Processing parser [44] with the biomedical domain model [45] to obtain the tokens, POS tags, and parse graphs for each word. Then, the phrase structure trees obtained by the parser are further processed using the Stanford conversion tool [46] to obtain the syntactic dependency graph.

The BB-rel and SeeDev-binary tasks are relation extraction tasks, which detect whether and what relations exist between 2 annotated entities in biomedical texts. For example, in the sentence “The percentage of penicillin-resistant *N. gonorrhoeae* isolated in the region over the decade varied considerably,” in which *N. gonorrhoeae* is a microorganism-type entity and “percentage” is a phenotype-type entity, we need to detect whether there is a relationship between them and the category of the relation. There are usually 2 solutions to the relation extraction task: the first is to identify whether there is a relation between entity pairs in a sentence and then classify a correct category [47], and the second method is to combine the 2 steps of identification and classification into 1 step [13]. This paper adopts the second method, which regards nonrelation as a category of relationships and carries out multi-category classification.

In the training and validation sets of the BB-rel and SeeDev-binary tasks, only positive instances were labeled. However, in the prediction phase, there may be a nonrelation between 2 candidate entities; therefore, it is necessary to manually construct negative instances in the training phase. After the biomedical texts are divided into sentences, we enumerate each entity pair in the sentence and judge the unlabeled instances as nonrelational. Because the biomedical relation extraction of SeeDev-binary and BB-rel tasks is under the constraint of regulation, there must be no relation between some entity types. For example, in the BB-rel task, there must be no biomedical relation between the entity of *geographic* type and the entity of *phenotype* type. Therefore, we need to further eliminate the entity pairs that do not comply with the regulations.

In the data sets of the 2 tasks, not only do the entities of a relation appear in the same sentence (intrasentence) but also the entities of a relation may be in different sentences (intersentence), which is a great challenge regarding biomedical relation extraction tasks [35]. In our method, we only considered intrasentence relations and ignored intersentence relations. There are 2 difficulties involved in the intersentence relation: one is that the reasoning relationship is difficult and complex; the other is that the number of negative instances increases exponentially, which leads to an extreme imbalance of positive and negative samples, resulting in performance degradation of the model. Therefore, all existing systems only extract intrasentence relations without considering intersentence relations [35,40]. In addition, an instance is eliminated if there is no syntactic dependency path between the 2 candidate entities.

### Distributed Semantic Representation

Our method extracts global semantic features from a full sentence through a combination of several distributed features and a multi-head attention mechanism. Domain-specific word embedding, POS embedding, entity-type embedding, distance embedding, and PE were integrated into our model.

Word embedding is a frequently used distributed representation model that encodes rich semantic information into vectors. The sequence of a full sentence of length *n* can be represented as *{w_1_,e_1_,...,e_2_,w_n_}*, where *e_1_* and *e_2_* represent entity pairs. We initialized our word embeddings with a pretrained 200-dimensional biomedical word embedding model [23], which was trained on PubMed and PMC abstracts, and full texts contained an unannotated corpus of 5 billion tokens. The pretrained embedding model was trained using the word2vec tool with the skip-gram model [48]. We only used the most frequent 100k words to build dictionary *D*, and the unknown words in the data sets were randomly initialized. Taking the BB-rel task as an example, it is possible that the words of entity are not in dictionary *D*, so we add the words “Microorganism,” “Habitat,” “Geographical,” and “phenotype” to the dictionary and initialize them randomly. If an entity is of *microorganism* type and is not in the word embedding model, it will be replaced by the word “Microorganism.” Through the pretrained word embedding matrix, we can transform the sequence of tokens in a full sentence into a vector sequence 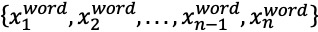. We also used POS embedding [24] to encode the POS for words in a sentence, which usually plays an important role. The POS embedding was randomly initialized and fine-tuned during the training phase.

The combination of different types of entities has different probabilities for some relations; therefore, the entity type is an important factor for prediction [13]. As the 2 biomedical relation extraction tasks are conditionally constrained, they do not involve the direction between entity pairs, so the entity-type sequence only needs one chain to represent. Therefore, the entity-type sequence can be expressed as {−1,*t_1_,...,t_2_,−1}*, where nonentity words are labeled as −1. Through a randomly initialized type embedding matrix, the entity-type vector sequence can be represented as 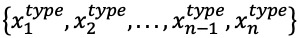.

The distance sequence is divided into 2 chains, namely, the distance from the current word to the 2 candidate entities. In our method, relative distance [25] is used to measure the distance between the current word and an entity, which can be formulated as equation 1, where *l* is the absolute distance and *s* is the maximum distance in the data sets. As the relative distance is not an integer, it is necessary to construct a distance dictionary and use the distance embedding matrix to generate the distance-vector sequence.



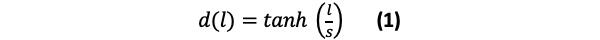



As we use the multi-head attention model to deal with the combination of a series of distributed features without using any time series model, we have to inject some absolute position information of words into the model; therefore, we introduce PE with reference as shown in the study by Vaswani et al [30]. In our method, the PE vectors have the same dimension *d_word_* as the word embedding, and then PE vectors can be calculated according to the sine and cosine functions of the frequencies. The formulas are given in equations 2 and 3, where *pos* is the position and *i* represents the *i*-th dimension of one word. Finally, the position information was injected into the model by adding the PE vector into the word embedding.



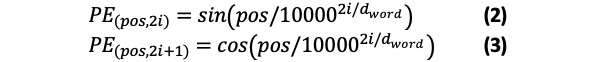



Finally, a series of distributed features is concatenated, and each word *w_i_* in the sentence can be represented as 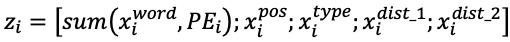. This comprehensive distributed feature is sent to the multi-head attention layer to extract the global semantic features of the full sentence.

### Multi-Head Attention Layer

In recent years, a series of attention-based models have been applied to relationship extraction tasks with remarkable success [49,50]. The core idea of the attention mechanism is to locate key information from text by assigning attention scores. At present, the most widely used attention models are additive attention [26] and dot-product attention [30]. In the study by Vaswani et al [30], the multi-head attention mechanism was proposed as the main component unit of the transformer model. In this model, attention can be used to compute the output of a series of values through value mapping to a set of key-value pairs, that is, to calculate a weighted sum of the values, where the weight assigned to each value is computed by a query with the corresponding key. In our method, the multi-head attention mechanism is used as an encoder to extract the global semantic feature of the full sentence, and each attention head is calculated by integrating the position information and using the scaled dot-product attention function.

**Figure 2:**
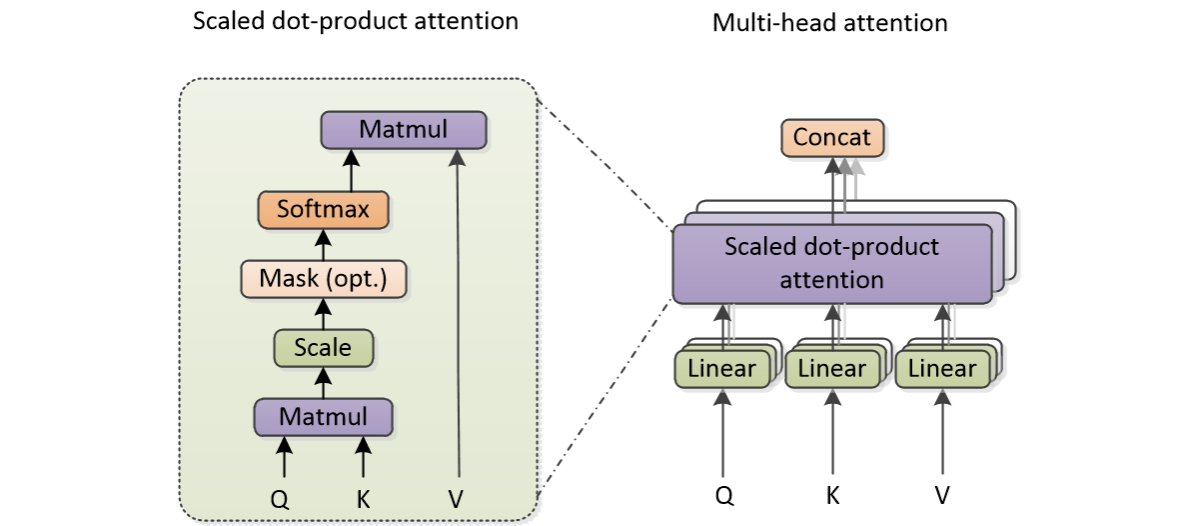
Scaled dot-product attention function (left). Multi-head attention consists of several scaled dot-product attention (right). Concat: concatenate; K: key; Matmul: matrix multiply; Q: query; V: value.

The overall structure of scaled dot-product attention and multi-head attention is shown in Figure 2, similar to that shown in the study by Vaswani et al [30]. Here, Q, K, and V are the same, which are the feature combinations from the full sentence; therefore, multi-head attention can also be understood as a form of self-attention. Eight attention heads based on scaled dot-product attention were used to extract features, which divided feature combinations into 8 channels. For each channel, the embedding of each word in the sentence with length *n* can be expressed as *z_i_*. Through the weights *(W_q,_ W_k_, W_v_)* that are not shared between channels, we can get the vector expression of a word in different subspaces, namely *(q_i_, k_i,_ v_i_),* as shown in equation 4.



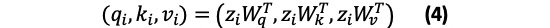



The attention weight vector *a_i_* corresponding to *i*-th query is calculated by the dot product of the query vector and key vector and then scaled by 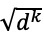 and calculated by a Softmax function, where *d^k^* is the dimensionality of the feature combination and *n* is the length of the sentence, as shown in equation 5.



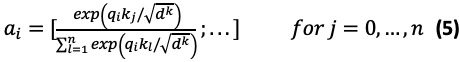



By multiplying the attention weight vector *a_i_* by the value sequence of length *n*, a feature vector *c_i_* is obtained, which is a weighted sum of the values, as shown in equation 6.



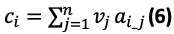



Therefore, the attention head of each channel is a concatenated matrix of *n* feature vectors, which can be expressed as *h_i_* using equation 7. Each attention head can encode the semantic information of a sentence in subspaces with different representations.

*h_i_* = [*c_1_;c_2_;...;c_n_*] **(7)**

Furthermore, we concatenated multiple attention heads in the last dimension to obtain the multi-head attention feature of the full sentence, as shown in equation 8.

*MultiHead* = [*h_1_;h_2_;...;h_8_*] **(8)**

Similar to the transformer model, we also used a fully connected neural network behind the multi-head attention model and used a residual join, as shown in Figure 1. Finally, the global semantic features of the full sentence are obtained using a max-pooling operation.

### Syntactic Dependency Feature

**Figure 3:**
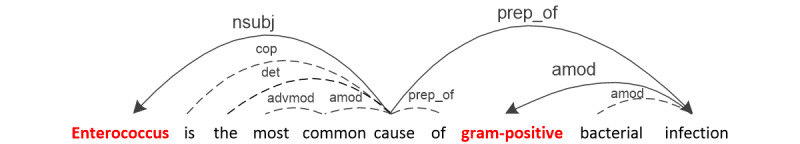
An example of syntactic dependency between phenotype-type entity “Enterococcus” and phenotype-type entity “Gram-positive”; solid lines are entity dependencies, and dashed lines are irrelevant dependencies. advmod: adverbial modifier; amod: adjectival modifier; cop: copula; det: determiner; nsubj: nominal subject; prep_of: preposition of.

The syntactic dependency features for the proposed DL model are generated based on the shortest dependency path connecting 2 candidate entities and the dependency type in the dependency graph. The shortest dependency path contains the most important terms related to characterizing the extraction and has been successfully applied in relation extraction many times [51,52]. An example of syntactic dependency is shown in Figure 3, where “Enterococcus” is a *microorganism-type* entity and “Gram-positive” is a *phenotype*-type entity. We can observe that the dependency parse between the words is directional. To simplify the calculation, we use the method by Mehryary et al [31] to convert the dependency relation of a sentence into an undirected graph and then find the shortest path between 2 candidate entities using the Dijkstra algorithm. In the case of BB-rel task, we always process from *a microorganism*-type entity to location entities (either a *habitat* or a *geographic* entity) or *phenotype* entity, regardless of their positions in sentences. Therefore, in the example in Figure 3, the shortest dependency path sequence is (“Enterococcus,” “cause,” “infection,” “Gram-positive”) and the dependency-type sequence is (nsubj, prep_of, amod).

In this case, the sequence of the shortest dependency path with *m* tokens can be represented as *{e_1_,w_2_,...,e_2_}*, where *e_1_* and *e_2_* represent the entity pairs at the head and end of the sequence, respectively. We used the previously mentioned pretrained 200-dimensional biomedical word embedding model [23]. Using the pretrained word embedding model, we can transform the dependent path sequence into a vector sequence 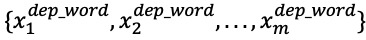. For the dependent-type sequence *{t_1_,t_2_,...,t_m−1_}*, we transform it into 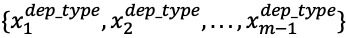 by randomly initializing the embedding matrix and filling it to the same length as the dependency path. The 2 vector sequences are concatenated, and *i*-th word can be denoted as 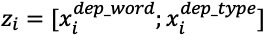.

To learn the local features of syntactic dependency from the dependency path and dependency type, LSTM [53] are the most frequently used DL models. By observing the length of the shortest dependency path, it is found that most of the interentity dependency lengths are 2 to 5, which belongs to the feature extraction of super-short sequences. Compared with LSTM, CNN is more suitable for super-short and concise sequences (Yin, W, unpublished data, February 2017). In addition, CNN are more suitable for parallel computing. Hence, we introduced a multifilter CNN model [54] and a max-pooling operation to learn syntactic dependency features, which has the advantage of learning hidden and advanced features from sentences with multiple channels.

### Classification and Training

In the output layer, we concatenate the global semantic feature vector and syntactic-dependent feature vector of the sentence to obtain a high-quality feature representation of the instance. Furthermore, the dropout algorithm [55] is used to prevent overfitting, the Softmax function is used to classify biomedical relations, and the probability distribution over each relation category is obtained.

The 2 tasks included a training set, validation set, and test set. In the training phase, taking the multi-classification cross entropy as the objective function, the Adaptive moment estimation optimization algorithm [56] with a learning rate of 0.001 was used to update the neural network parameters. The training times determine the generalization performance of the model; that is, too few training epochs lead to underfitting, and overtraining leads to overfitting. Therefore, the traditional early stopping method is adopted in our method, that is, training is stopped when the performance on the validation set is no longer improved. The experimental results show that the training epoch number is not a fixed value and that the model generally converges in approximately 4 epochs.

The data sets of the 2 biomedical relation extraction tasks were relatively small, and the DL model had more training parameters. Consequently, the initial random state of the model may have a significant impact on the final performance of the model, which was verified by a pre-experiment. To reduce the impact of the initialization state on the model, 10 different random initializations were used to evaluate the model, which was to train the same model structure with different random seeds. Finally, the model with the best *F*_1_ score on the validation set was used as the final model. We used the final model to predict the test set and used the results to evaluate our model on a web-based evaluation service.

### Parameter Settings

Through the pre-experiment and evaluation based on the validation set, the hyperparameters of our model were determined. The dimensions of domain-specific word embedding, POS embedding, entity-type embedding, distance embedding, PE, and dependency-type embedding were 200, 200, 200, 100, 200, and 200, respectively, and the embedding matrix was fine-tuned during the training phase. For the multi-head attention mechanism, we adopted a single-layer multi-head attention model, in which 8 parallel attention heads were used, and the number of units in the linear layer of each attention head was the same as the input. To extract the syntactic dependency feature, the number of convolution layers was 1, the number of filters was set to 128, and the window sizes were 2, 3, and 4. In addition, the LSTM model was used in the experiment, and the output dimension of the hidden units was set as 128. For the combination of global semantic features and syntactic dependency features, the dropout rate was 0.5. The batch size was set to 8. Finally, we used the DL framework Pytorch [57] to implement our model and carry out the experimental process.

### Ethics Approval

The data set and methods used in this work are publicly available and do not involve any ethical or moral issues.

## Results

### Data Set and Evaluation Metrics

We conducted a series of experiments on the BB-rel and SeeDev-binary task data sets to evaluate our proposed approach.

The BB-rel task in BioNLP-OST 2019 is quite different from the previous versions, which integrate the new entity type of *phenotype* and relation category of *Exhibits*. Therefore, this task involves 4 entity types, *microorganism*, *habitat*, *geography*, and *phenotype*, and 2 relation categories between entity pairs, *Lives_In* and *Exhibits*. In practice, the nonrelation between entity pairs is also regarded as a prediction category, so this task is treated as a multi-classification relation extraction task. In addition to intrasentence relations, the BB-rel task also considers intersentence relations, which remains a significant challenge. The proportion of intersentence relationships in the corpus was 17.5%. In our method, we consider only the intrasentence relationship. We adopted the method described in the data preprocessing section to segment the text into sentences, construct negative instances, and remove instances that do not comply with the constraint of regulation. In this manner, we constructed 1996 training instances, including 943 related instances; 1040 validation instances, including 517 related instances; and 1414 test instances. The detailed distribution of the BB-rel task data set after the preprocessing procedure is summarized in Table 1. Owing to different data revision and processing methods, the number of instances may be inconsistent with other studies.

We used the predictions of the test set to evaluate our methods on the web-based evaluation service [58]. Its evaluation metrics are similar to those of previous versions, including precision, recall, *F*_1_ score, and the results of the intrasentence and intersentence relations of various relation categories [35].

The SeeDev-binary task corpus is a set of 87 paragraphs from 20 full articles on the seed development of *Arabidopsis thaliana*, with 17 entity types and 22 relation categories manually annotated by domain experts. There are 3575 annotated relations, including 1628 relations for the training sets, 819 relations for the validation sets, and 1128 relations for the test sets. We used the same method to preprocess the data set and eliminate intersentence relations. Then, 18,997 training instances were constructed, including 1508 related instances; 8955 validation instances were constructed, including 746 related instances; and 12,737 test instances were constructed, and the detailed distribution is shown in Table 2. It can be seen that there is an extreme imbalance where the number of nonrelation samples far exceeds the positive samples, which is more challenging and will negatively affect the performance of the model [47]. Therefore, to alleviate this problem, through a series of pre-experiments, we finally decided to randomly delete 90% (15,740/17,489) of the negative samples in the training stage, but the validation and test sets were not reduced.

The SeeDev-binary is also applicable to the web-based evaluation services. Compared with SeeDev-binary 2016, task organizers have added new evaluation metrics to emphasize biomedical contributions. The evaluation metrics are global results for all relations, the results of intrasentence relations, and type clusters, each of which has a precision, recall, and *F*_1_ score.

**Table 1:** Detailed statistics of the relation extraction of Bacteria-Biotope task data set. The statistics of the test set is none because the organizer has not released the annotated relation on the test set.

Category	Training set	Validation set	Test set
Total	1996	1040	1414
Lives_in	659	377	None
Exhibits	284	140	None
Lives_in and Exhibits	943	517	None
Nonrelation	1053	523	None

**Table 2:** Detailed statistics of the binary relation extraction of plant seed development task data set. The number of relationships in the test set is none because the number of relationships cannot be determined after preprocessing.

Category	Training set	Validation set	Test set
Total	18,997	8955	12,737
All relation	1508	746	None
Nonrelation	17,489	8209	None

### Experiment Results

In the BB-rel task, we used the proposed DL model based on the multi-head attention mechanism and syntactic dependency feature to detect biomedical relations. Our proposed method finally obtained an *F*_1_ score of 65.56% on the test set; the details are shown in Table 3. Our method has an *F*_1_ scores of 62.36% and 73.62% for the relation category of *Lives_In* and *Exhibits*, respectively, and performs better in the relation category *Exhibits*. Moreover, it can be noted that the *F*_1_ scores in the identification of intrasentence relations of Lives_In and Exhibits are 69.00% and 77.67%, which are higher than the comprehensive *F*_1_ score. This is because our preprocessing method only deals with intrasentence relations; therefore, it performs better in the identification of intrasentence relations.

Table 4 lists the comparison between our method and other previous systems in BB-rel task. The first 3 lines in the table are the official top 3 systems (10 participated), among which Yuhang_Wu used a multilayer perceptron [35], AliAI [39] used a multitask architecture similar to BERT, and whunlp [37] achieves state-of-the-art performance by using dependency graph and attention graph convolution neural network. The fourth line is the baseline provided by the task organizer, which uses a co-occurrence method. Owing to the huge difference between the model architecture of these systems, only the final *F*_1_ score is used for comparison. The *F*_1_ score of our method is 5.07% higher than the third-placed Yuhang_Wu and 0.60% superior to the second-placed AliAI, who achieved the result of 64.96%. It is worth noting that our model achieved the best precision of 69.50%, which is superior to all existing systems in BB-rel task. This result reveals that our method tends to predict fewer positive classes, that is, it performs better on false positives than other models. In conclusion, this comparison indicates that our proposed model is effective and achieved excellent performance in BB-rel task.

In the SeeDev-binary task, our proposed method achieved an *F*_1_ score of 38.04% for all relations in the test set. The detailed results for the specific relation categories are shown in Table 5. As shown in the table, 7 types of relation categories were not detected, such as *Is_Involved_In_Process* and *Occurs_During*. Through the statistical analysis of the data set, it was found that there were few positive instances of these relation categories in the training set, which was obviously responsible for the uneven classification.

Table 6 lists the results of comparison between our method and other systems for the SeeDev-binary task. The first 2 systems are the top 2 of the official ranks in BioNLP-OST 2019. Among them, the first-placed MIC-CIS [42] used linguistic feature and SVM classifier to achieve an *F*_1_ score of 37.38%, whereas YNU-junyi [14], the second-ranking system, obtained an *F*_1_ score of 34.18% using a DL model combined with distributed representation, CNN and LSTM model. The results show that our method achieves the state-of-the-art performance in both category of all relation and intrasentence relation, with *F*_1_ scores of 38.04% and 38.68%, respectively. In the all-relation category, the *F*_1_ score of our system outperformed the first-ranking system by 0.66% and the second-ranking system by 3.86%. Meanwhile, the result is similar to BB-rel task; our system performed excellently in precision. In All relation and intrasentence relation, the precision surpassed the first-ranking system by 7.30% and 5.30%, respectively. This once again proves that our model has a lower false-positive rate than other models. Therefore, we can conclude that our model can take advantage of both the multi-head attention mechanism and syntactic dependency feature to achieve excellent performance in biomedical relation extraction tasks.

The results by cluster are also important evaluation metrics in the SeeDev-binary task, and the comparison of *F*_1_ scores is shown in Table 7. It can be seen from the table that our model achieves optimal results in 3 cluster categories: *function*, *regulation*, and *genic regulation,* and it performs poorly in 2 cluster categories: *composition membership* and *interaction*, but the overall performance of our proposed model is generally satisfactory.

**Table 3:** Detailed results of our method on the test set of relation extraction of Bacteria-Biotope task.

Category	Precision	Recall	*F*_1_ score
Lives_In and Exhibits	69.50	62.05	*65.* *56* ^a^
Lives_In	69.38	56.64	62.36
Lives_In (intrasentence)	69.75	68.27	69.00
Exhibits	69.77	77.92	73.62
Exhibits (intrasentence)	70.18	86.96	77.67

^a^The final *F*_1_ score is shown in italics.

**Table 4:** Comparison of results between our method and other systems for the relation extraction of Bacteria-Biotope task.

Models	Precision	Recall	*F*_1_ score
whunlp [37]	62.94	*70.22* ^a^	*66.38*
AliAI [39]	68.20	62.01	64.96
Yuhang_Wu [35]	55.10	67.03	60.49
Baseline [35]	52.54	80.13	63.47
Our model	*69.50*	62.05	65.56

^a^The maximum results are shown in italics.

**Table 5:** Detailed results of our method on the test set of the binary relation extraction of plant seed development task.

Binary relation type	Precision	Recall	*F*_1_ score
Exists_In_Genotype	40.59	32.28	35.96
Occurs_In_Genotype	0	0	0
Exists_At_Stage	50.00	10.00	16.67
Occurs_During	0	0	0
Is_Localized_In	38.16	46.77	42.03
Is_Involved_In_Process	0	0	0
Transcribes_Or_Translates_To	0	0	0
Is_Functionally_Equivalent_To	60.94	55.71	58.21
Regulates_Accumulation	66.67	25.00	36.36
Regulates_Development_Phase	22.86	41.56	29.49
Regulates_Expression	24.65	50.72	33.18
Regulates_Molecule_Activity	0	0	0
Regulates_Process	40.04	64.71	49.47
Regulates_Tissue_Development	0	0	0
Composes_Primary_Structure	60.00	37.50	46.15
Composes_Protein_Complex	50.00	66.67	57.14
Is_Protein_Domain_Of	26.09	19.35	22.22
Is_Member_Of_Family	27.78	52.33	36.29
Has_Sequence_Identical_To	100.00	47.73	64.62
Interacts_With	80.00	14.81	25.00
Binds_To	30.77	12.50	17.78
Is_Linked_To	0	0	0
All relations	34.75	42.02	*38.04* ^a^

^a^The final *F*_1_ score is shown in italics.

**Table 6:** Comparison of results between our method and other systems for the binary relation extraction of plant seed development task.

Models	All relation				Intrasentence relation		
	Precision	Recall	*F*_1_ score	Precision		Recall	*F*_1_ score
MIC-CIS [42]	27.45	*51.15* ^a^	37.38	29.45		*53.08*	37.88
YNU-junyi [14]	27.25	45.83	34.18	27.25		47.56	34.65
Our method	*34.75*	42.02	*38.04*	*34.75*		43.61	*38.68*

^a^The maximum results are shown in italics.

**Table 7:** Comparison of *F*_1_ scores by cluster between our method and other systems for the binary relation extraction of plant seed development task.

Models	All	Comparison	Function	Regulation	Genic regulation	Composition membership	Interaction
MIC-CIS [42]	37.38	47.92	17.39	34.78	33.84	*40.25* ^a^	*34.24*
YNU-junyi [14]	34.18	*50.45*	25.00	34.21	23.00	34.68	21.87
Our method	*38.04*	49.68	*25.53*	*40.78*	*34.04*	32.72	22.02

^a^The maximum results are shown in italics.

## Discussion

### Overview

In this section, we construct ablation experiments to analyze the effectiveness of multi-head attention mechanism and syntactic dependency feature. To avoid the instability of a single model, the mean *F*_1_ score on the test set was used to measure model performance. Subsequently, we conducted an error analysis and manually analyzed the correct and incorrect predictions.

### Effectiveness of Multi-Head Attention Mechanism

We first analyzed the effectiveness of the multi-head attention mechanism in the global semantic feature extraction of a full sentence compared with the traditional CNN, BiLSTM, and additive attention models [26]. All models use the distributed features and syntactic dependency features that we use, such as domain-specific word embedding. Owing to the application of PE in the multi-head attention mechanism, we integrate PE into all models for a fair comparison. Table 8 shows a comparison of the mean *F*_1_ scores using various models to encode global semantic features.

From the table, the first 2 lines are the results of extracting the feature representation of sentences using the CNN or BiLSTM model alone, among which the result of the BiLSTM model was slightly better. A possible explanation is that the length of sentences in instances is generally large, and the CNN model can only process window information and rely on a pooling operation to summarize the overall structure of the sentences. However, the BiLSTM model is more suitable for sequence modeling and encoding longer sequence information using a bidirectional memory network. They were then combined with an additive attention model. Compared with CNN and LSTM models alone, the application of the attention model improved *F*_1_ scores by 1.82% and 1.22% on BB-rel and 1.31% and 1.11% on SeeDev-binary, respectively. In addition, the performance of CNN with attention exceeds that of BiLSTM with attention on the BB-rel task, possibly because the attention mechanism fills the shortcoming that CNN cannot capture the long-range dependence of sentences. Hence, these results suggest that the attention mechanism can effectively improve the performance of the model by focusing on the key information of the token sequence and learning the overall structure of a sentence.

Finally, the multi-head attention mechanism is introduced into our model without any CNN or recurrent neural network structure, and the optimal result is achieved. The mean *F*_1_ score was 63.13% and 36.37% for the 2 tasks, which are 1.11% and 1.24% higher than that of the BiLSTM-attention model and 0.96% and 1.45% higher than that of the CNN-attention model, respectively. The results show that the multi-head attention mechanism significantly outperforms the additive attention model in biomedical relation extraction. To some extent, additive attention can be understood as a single-head attention model that can only learn the global semantic features in one representation space. However, the advantage of the multi-head attention mechanism is that it captures the global semantic information in different representation subspaces and integrates the contextual information of relevant words into the current word from multiple channels. The experimental results demonstrate that the multi-head attention mechanism can extract more comprehensive feature representations and effectively improve the performance of the relation extraction model.

**Table 8:** The comparison of mean *F*_1_ score of using different models to extract global semantic features in the relation extraction of Bacteria-Biotope task (BB-rel) and the binary relation extraction of plant seed development task (SeeDev-binary).

Global semantic features	BB-rel			SeeDev-binary		
	Minimum^a^	Maximum^b^	Mean (SD)	Minimum^a^	Maximum^b^	Mean (SD)
CNN^c^	57.26	63.26	60.35 (2.11)	31.67	35.85	33.61 (1.33)
BiLSTM^d^	57.89	63.80	60.80 (1.88)	32.39	36.28	34.02 (1.53)
CNN-attention	59.69	65.01	62.17 (1.69)	32.89	37.52	34.92 (1.47)
BiLSTM-attention	59.80	64.38	62.02 (1.45)	33.61	37.30	35.13 (1.18)
Multi-head attention	*60.68* ^e^	*65.56*	*63.13 (* *1.55* *)*	*34.47*	*38.04*	*36.37 (1.13)*

^a^The lowest *F*_1_-scores of 10 different random initializations.

^b^The highest *F*_1_-scores of 10 different random initializations.

^c^CNN: convolutional neural network.

^d^BilSTM: bidirectional long short-term memory network.

^e^The maximum results are shown in italics.

### Effectiveness of Syntactic Dependency Feature

Furthermore, we analyzed the effectiveness of the syntactic dependency feature in our model. The length of the shortest dependency paths, based on syntactic analysis, is mostly 2 to 5, which belongs to a super-short sequence. Therefore, we only tried to use the CNN and BiLSTM models for feature extraction, and the results are shown in Table 9. The first line shows the results that the model does not use syntactic dependency features, and the average *F*_1_ scores were 60.85% and 34.60% for BB-rel and SeeDev-binary tasks, respectively. When the LSTM model was used to extract syntactic dependency features, the mean *F*_1_ scores of the model were 62.88% and 36.06%. When we used the CNN model, the performance of the model reached optimal *F*_1_ scores, which improved to 63.13% and 36.37% on BB-rel and SeeDev-binary tasks, respectively. The results also show that the CNN model is superior to LSTM in terms of feature extraction for super-short sequences. By comparison, it can be demonstrated that the integration of syntactic dependency features can enable the model to learn syntactic information between entity pairs through a dependency graph, which can effectively improve the performance of the model.

**Table 9:** The comparison of mean *F*_1_ scores of using different models to extract syntactic dependency features in the relation extraction of Bacteria-Biotope task (BB-rel) and the binary relation extraction of plant seed development task (SeeDev-binary).

Syntactic dependency feature	BB-rel			SeeDev-binary		
	Minimum^a^	Maximum^b^	Mean (SD)	Minimum^a^	Maximum^b^	Mean (SD)
No-use	58.51	63.70	60.85 (1.65)	32.89	36.53	34.60 (1.16)
LSTM^c^	59.93	65.16	62.88 (1.66)	*34.55* ^d^	37.90	36.06 (1.07)
CNN^e^	*60.68*	*65.56*	*63.13 (1.55)*	34.47	*38.04*	*36.37 (1.13)*

^a^The lowest *F*_1_-scores of 10 different random initializations.

^b^The highest *F*_1_-scores of 10 different random initializations.

^c^LSTM: long short-term memory network.

^d^The maximum results are shown in italics.

^e^CNN: convolutional neural network.

### Error Analysis

To verify the advantages and weaknesses of our proposed model, we compared the experimental results with those of other existing models. We find that our system performs better in terms of the precision of the 2 relation extraction tasks, far surpassing other models, which means that our approach has a lower false-positive rate than the other models. One possible explanation is that our model structure introduces the shortest dependent paths compared with other systems, which can more definitely identify the biomedical relationship between entity pairs.

The 2 relationship extraction tasks are constrained under regulations; therefore, it is necessary to investigate whether there is a situation in which the predicted relationship does not conform to the rules. For example, in the sentence “An evaluation of selective broths based on the bi-selenite ion and on hypertonic strontium chloride in *Salmonellae* detection in egg products,” the entity “Salmonellae” is of *microorganism* type, and the entity “egg products” is of *habitat* type. There may be a *Lives_In* relationship between them, but if it is predicted as *an Exhibits* relationship, it must be wrong. Through an analysis of the prediction results on the validation set, it was found that this situation rarely occurs. Therefore, our research should focus on whether a biomedical relationship exists between entity pairs.

In addition, we manually analyzed the correct and false predictions from the validation set compared with existing DL models (structures similar to YNU-junyi [14]). We found that our proposed model generally performed better on long sentences. A complicated sentence structure and long distance between 2 entities are more likely to lead to relationship classification errors. For example, in the sentence “The prevalence of H. pylori infection in dyspeptic patients in Yemen is very high, the eradication rate with standard triple therapy was unsatisfactory probably because of widespread bacterial resistance due to unrestricted antibiotic use,” “H. pylori” is a *microorganism* entity, “widespread bacterial resistance due to unrestricted antibiotic use” is *a phenotypic* entity, and there is an *Exhibits* relationship between them. The DL model, similar to YNU-junyi, predicted it as a nonrelationship category, but our model can better detect it, probably because our proposed model can capture the long-term dependency between words in a long sentence.

### Conclusions

This paper focuses on the 2 relation extraction tasks in BioNLP-OST 2019: BB-rel task and SeeDev-binary task, which aim to promote the development of fine-grained IE from biomedical texts. For these tasks, we propose a DL model based on the combination of a series of distributed features to detect relations, introduce a multi-head attention mechanism to extract global semantic features, and use syntactic-dependent features to enrich the feature representation. Our proposed method obtained *F*_1_ scores of 65.56% and 38.04% on the test sets of the 2 tasks and achieved state-of-the-art results in the SeeDev-binary task. Through ablation experiments, the effectiveness of multi-head attention and syntactic dependency features was demonstrated. The multi-head attention mechanism allows the model to learn relevant semantic information in different representation subspaces at different positions and integrates the contextual information of relevant words in the sentence into the current word representation, which greatly improves the performance of the biomedical relation extraction model.

Despite the excellent performance of our model on BB-rel and SeeDev-binary tasks, there are still many challenges. In particular, the intersentence relation is not considered in our method, which remains a difficult problem in biomedical relation extraction tasks. This situation is because of the complexity of the reasoning relationship and the extreme imbalance between the positive and negative examples. In contrast, the use of a DL model to extract high-quality features from small training data sets is a problem that needs to be solved. In future work, we will consider using a semisupervised learning method or transformer model, such as BERT, to better solve the topic of biomedical relation extraction.

## Abbreviations

BB-rel: relation extraction of Bacteria-Biotope task
BERT: Bidirectional Encoder Representations from Transformers
BiLSTM: bidirectional long short-term memory
BioNLP-OST: Biomedical Natural Language Processing Workshop-Open Shared Task
CNN: convolutional neural network
DL: deep learning
IE: information extraction
LSTM: long short-term memory
PE: position embedding
POS: part of speech
SeeDev-binary: binary relation extraction of plant seed development task
SVM: support vector machine
TEES: Turku Event Extraction System
